# Undergraduate nursing and midwifery student's attitudes to mental illness

**DOI:** 10.1002/nop2.494

**Published:** 2020-04-14

**Authors:** Angela Hawthorne, Ross Fagan, Elspeth Leaver, Jessica Baxter, Pamela Logan, Austyn Snowden

**Affiliations:** ^1^ School of Health and Social Care Edinburgh Napier University Edinburgh UK; ^2^ Rapid Response Team, Royal Edinbugh Hospital Edinburgh UK; ^3^ South East Recovery Hub South Neighbourhood Office (West Wing) Edinburgh UK; ^4^ HMP Addiewell Addiewell UK; ^5^ Huntlyburn Ward Borders General Hospital Melrose UK

**Keywords:** attitudes, exposure, mental illness, nurses, stigma, student

## Abstract

**Aim:**

To explore levels of stigma in students of all fields of nursing and midwifery at different years and examine the impact of exposure to people with mental illness.

**Design:**

A cross‐sectional survey was used.

**Methods:**

The Community Attitudes to Mental Illness questionnaire was administered to all branches of student nurses (adult health, mental health, child health and learning disability) and midwives in all three years in one Higher Education Institution (HEI) in Scotland.

**Results:**

Mental health nursing students scored significantly better on all stigma subscales. Stigma worsened with a little professional exposure to people with mental illness but then improved with increasing exposure. Both personal exposure and professional exposure to people with mental illness change perceptions. The professional results follow a J‐curve. Current plans for cross‐field experience involving short or virtual placements during student nurse training are likely to worsen stigma rather than improve it.

## 1 INTRODUCTION

Public or “external” stigma consists of three key elements*: Stereotypical negative beliefs about a group (e.g. dangerousness, incompetence, character weakness); Prejudiced agreement with a belief and/or negative emotional reaction (e.g* *. anger, fear, disgust); and Discriminating behavioural responses to the targets of prejudice (e.g. avoidance, employment and housing discrimination, reluctance to help (*Corrigan & Watson, 2002, p. 16).

Self‐stigma is the internalization of these stereotypical views and behaviours (Berry & Greenwood, 2017). Public stigma can create self‐stigma, which can in turn lead people with severe mental illness to having feelings of hopelessness and despair, which can significantly impair recovery (Yanos, Roe, Markus, & Lysaker, 2008). However, it is possible to mediate the impact of stigma by reinforcing personal attributes such as resilience (Sickel, Seacat, & Nabors, 2016). This paper examines levels of stigma in a student nurse cohort. For the purpose of this article, the term “mental illness” is used due to its consistency with the measurement tool used in the study.

Stigma has been studied internationally in relation to mental health nursing, both in terms of current practice and in terms of education settings. In the United Kingdom, Schafer, Wood, and Williams (2011) compared attitudes to mental illness of adult health and mental health students in their second week of training. They found that mental health nursing students had significantly more positive attitudes than adult health nursing students. There have been numerous studies conducted on nursing attitudes towards mental illness in Australia, where undergraduate nursing degrees are more general focused, with specialization into different areas such as mental health taking place at graduate level (Happell, 2008, 2009; Happell & Gaskin, 2012; Happell & Gough, 2009). The overall thesis from such research is that nurses stigmatize people with mental illness in the same way the general public do. Annear, Lea, Lo, Tierney, and Robison (2016) studied the effects of reducing stigma towards elderly people in medical students. The study concluded that by introducing a short clinical placement into the educational programme, it can improve medical students’ attitudes towards working with elderly people and dementia care. It has also shown that attitudes can be changed through education. For example, Happell and Gaskin (2012) showed that mental health nursing was initially one of the least preferred areas of work for undergraduate nurses. They hypothesize that more education and more clinical placements would improve attitudes towards people with mental illness.

Gillespie (2013) suggested that there needs to be a change in nursing education to facilitate a change in attitudes as well as levels of compassion towards people with long‐term conditions. Gillespie (2013) concluded that students currently viewed working in long‐term units (and especially non‐NHS care settings) as to be less fulfilling and less attractive than working in acute units. Therefore, there is a potential for change in the undergraduate programmes for nursing students where a shift in education and clinical practice placements may allow for nursing students to have more exposure towards people with long‐term conditions with the aim to increase the levels of compassion and reduce stigma.

Thongpriwan et al. (2015) tested this idea in the United States. They examined whether stigma could be mitigated by “educational preparation,” through either clinical practice or classroom‐based learning. They found positive results, with students undergoing the training feeling more prepared for the role of being a mental health nurse. Successful studies such as this are important because stigma can have a detrimental effect on the nurse–patient relationship (Delaney, 2012). If stigma can be reduced, then it is reasonable to suggest that people could recover more quickly. It has been understood for a long time that stigma is one of the biggest barriers to recovery (Caltaux, 2002).

Every nurse, whether adult health, child health, learning disability or midwife will at some point likely care for a person who has a comorbid mental illness in additon to what thay are being directly treated for. It would benefit the recipient of care greatly if stigma could be eliminated. Unfortunately, most of the literature suggests that nurses in general medical settings often have the most negative attitudes towards people with mental illness (Arvaniti et al., 2009; Rao et al., 2009; Ross & Goldner, 2009). These attitudes need to be challenged and so it would be worthwhile understanding where they originate. Exploring the attitudes of undergraduate nursing and midwifery students is key in starting to understand where these attitudes arise and what, if anything, can be done to challenge them.

This study repeats Schafer et al.'s (2011) methods but extends the participant group from just adult and mental health nursing students to examine all fields of undergraduate nursing and midwifery practice in the UK: adult, mental health, child, learning difficulties and midwifery. Schafer et al. (2011) used the Community Attitudes towards Mental Illness scale (CAMI Scale). Taylor & Dear (1981) developed the CAMI scale in the 1980s as a response to the growing move towards care in the community at that time. The aim of the study was to explore levels of stigma in all fields of nursing and midwifery, at different years of study, and to examine the impact of exposure to people with mental illness, this is the only study in Britain to look at all branches of nursing over the full course. There were three main hypotheses:

Hypothesis 1 Different branches of nursing and midwifery will have different attitudes towards mental illness.

Hypothesis 2 Previous exposure to people with mental illness will influence attitudes.

Hypothesis 3 Each branch of nursing and midwifery students will have different attitudes towards mental illness throughout first year, second year and third year.

## 2 METHOD

### 2.1 Design

Cross‐sectional survey design.

### 2.2 Participants

All student nurses and midwifery students at a large HEI in Scotland.

### 2.3 Data collection

Following Schafer et al. (2011), attitudes were measured using the CAMI Scale. This questionnaire has 40 items designed to measure attitudes on four subscales: authoritarianism, benevolence, social restrictiveness and community mental health ideology. The prereliability–postreliability (Pearson = 0.87) and internal consistency (Cronbach's Alpha = 0.898) of the CAMI have been tested and found to be acceptable (Schafer et al., 2011). The full scale is in Table 1.

**Table 1:** The Community Attitudes towards Mental Illness scale

The following statements express various opinions about mental illness and the mentally ill. The mentally ill refers to people needing treatment for mental disorders but who are capable of independent living outside a hospital				
Please circle the response which most accurately describes your reaction to each statement. It's your first reaction which is important. Don't be concerned if some statements seem similar to ones you have previously answered. Please be sure to answer all statements				
a. As soon as a person shows signs of mental disturbance, he should be hospitalized				
SA	A	N	D	SD
b. More tax money should be spent on the care and treatment of the mentally ill				
SA	A	N	D	SD
c. The mentally ill should be isolated from the rest of the community				
SA	A	N	D	SD
d. The best therapy for many mental people is to be part of a normal community				
SA	A	N	D	SD
e. Mental illness is an illness like any other				
SA	A	N	D	SD
f. The mentally ill are a burden on society				
SA	A	N	D	SD
g. The mentally ill are far less of a danger than most people suppose				
SA	A	N	D	SD
h. Locating mental health facilities in a residential area downgrades the neighbourhood				
SA	A	N	D	SD
i. There is something about the mentally ill that makes it easy to tell them from normal people				
SA	A	N	D	SD
j. The mentally ill have for too long been the subject of ridicule				
SA	A	N	D	SD
k. A woman would be foolish to marry a man who has suffered from mental illness, even though he seems fully recovered				
SA	A	N	D	SD
l. As far as possible mental health services should be provided through community based facilities				
SA	A	N	D	SD
m. Less emphasis should be placed on protecting the public from the mentally ill				
SA	A	N	D	SD
n. Increased spending on mental health services is a waste of tax dollars				
SA	A	N	D	SD
o. No one has the right to exclude the mentally ill from their neighbourhood				
SA	A	N	D	SD
p. Having mental people living within residential neighbourhoods might be good therapy, but the risks to residents are too great				
SA	A	N	D	SD
q. Mental people need the same kind of control and discipline as a young child				
SA	A	N	D	SD
r. We need to adopt a far more tolerant attitude toward the mentally ill in our society				
SA	A	N	D	SD
s. I would not want to live next door to someone who has been mentally ill				
SA	A	N	D	SD
t. Residents should accept the location of mental health facilities in their neighbourhood to serve the needs of the local community				
SA	A	N	D	SD
u. The mentally ill should not be treated as outcasts of society				
SA	A	N	D	SD
v. There are sufficient existing services for the mentally ill				
SA	A	N	D	SD
w. Mental people should be encouraged to assume the responsibilities of normal life				
SA	A	N	D	SD
x. Local residents have good reason to resist the location of mental health services in their neighbourhood				
SA	A	N	D	SD
y. The best way to handle the mentally ill is to keep them behind locked doors				
SA	A	N	D	SD
z. Our mental hospitals seem more like prisons than like places where the mentally ill can be cared for				
SA	A	N	D	SD
aa. Anyone with a history of mental problems should be excluded from taking public office				
SA	A	N	D	SD
bb. Locating mental health services in residential neighbourhoods does not endanger local residents				
SA	A	N	D	SD
cc. Mental hospitals are an outdated means of treating the mentally ill				
SA	A	N	D	SD
dd. The mentally ill do not deserve our sympathy				
SA	A	N	D	SD
ee. The mentally ill should not be denied their individual rights				
SA	A	N	D	SD
ff. Mental health facilities should be kept out of residential neighbourhoods				
SA	A	N	D	SD
gg. One of the main causes of mental illness is a lack of self‐discipline and will power				
SA	A	N	D	SD
hh. We have the responsibility to provide the best possible care for the mentally ill				
SA	A	N	D	SD
ii. The mentally ill should not be given any responsibility				
SA	A	N	D	SD
jj. Residents have nothing to fear from people coming into their neighbourhood to obtain mental health services				
SA	A	N	D	SD
kk. Virtually anyone can become mentally ill				
SA	A	N	D	SD
ll. It is best to avoid anyone who has mental problems				
SA	A	N	D	SD
mm. Most women who were once people in a mental hospital can be trusted as baby sitters				
SA	A	N	D	SD
nn. It is frightening to think of people with mental problems living in residential neighbourhoods				
SA	A	N	D	SD

Community attitudes toward the mentally ill					
Key to items	Scoring				
	SA	A	N	D	SD
Authoritarianism					
Pro: a,i,q,y,gg	5	4	3	2	1
Anti: e,m,u,cc,kk	1	2	3	4	5
Benevolence					
Pro: b,j,r,z,hh	5	4	3	2	1
Anti: f,n,v,dd,ll	1	2	3	4	5
Social Restrictiveness					
Pro: c,k,s,aa,ii	5	4	3	2	1
Anti: g,o,w,ee,mm	1	2	3	4	5
Community Mental Health Ideology					
Pro: d,l,t,bb,jj	5	4	3	2	1
Anti: h,p,x,ff,nn	1	2	3	4	5

Abbreviations: A, Agree; D, Disagree; N, Neutral; SA, Strongly Agree; SD, Strongly Disagree.

In addition to the CAMI, demographic data were collected from participants as well as information about their previous exposure to mental illness in both a personal capacity and a professional capacity. Participants were asked to rate their previous personal and professional experience of mental illness on a Likert scale ranging from 0, meaning no experience, to 5, meaning a lot of experience. Personal experience might have been gained through their own experiences or those of family and friends, while professional experience might have been gained through university placements and previous employment. Participants were also asked whether they felt that overall their previous experience of mental illness had been positive, negative or neutral.

### 2.4 Process

The CAMI questionnaire was distributed to all undergraduate nursing and midwifery students at a single HEI in Scotland (*N* = 1,800). The study commenced in January 2016, and data collection was carried out over 3 months. Participation in the study was open to all students. Inclusion criteria for participation were that they must be 18 years or older and enrolled in an undergraduate nursing or midwifery course at that single HEI in Scotland. Posters were displayed around the university, and emails were sent to lecturers telling them about the research and asking to arrange a time to come and speak to their students. At the arranged time, the students were told about the research and those who were interested were given the chance to participate. The four core researchers were also all students at the university. Data were collected from students in all 3 years of study.

### 2.5 Ethics

Permission to carry out the study was given by the Edinburgh Napier University School of Health and Social Care Ethics Committee. All participants were provided with information about the study prior to taking part, were told that their data would be analysed anonymously, signed consent forms and had the option to opt out at any time.

### 2.6 Analytic plan

A power analysis was completed prior to commencement of the study which indicated that a sample size of 433 would be desirable. The data collected from the questionnaires were entered into SPSS version 20. For hypothesis 1, the groups were checked for normality and homogeneity of variance before running either ANOVA or relevant non‐parametric equivalent, testing each subscale in the CAMI against each branch of nursing. For hypotheses 2 and 3, the same assumptions were checked before running the relevant parametric or non‐parametric tests (Lund & Lund, 2015).

## 3 FINDINGS

### 3.1 Sample

**Figure 1:**
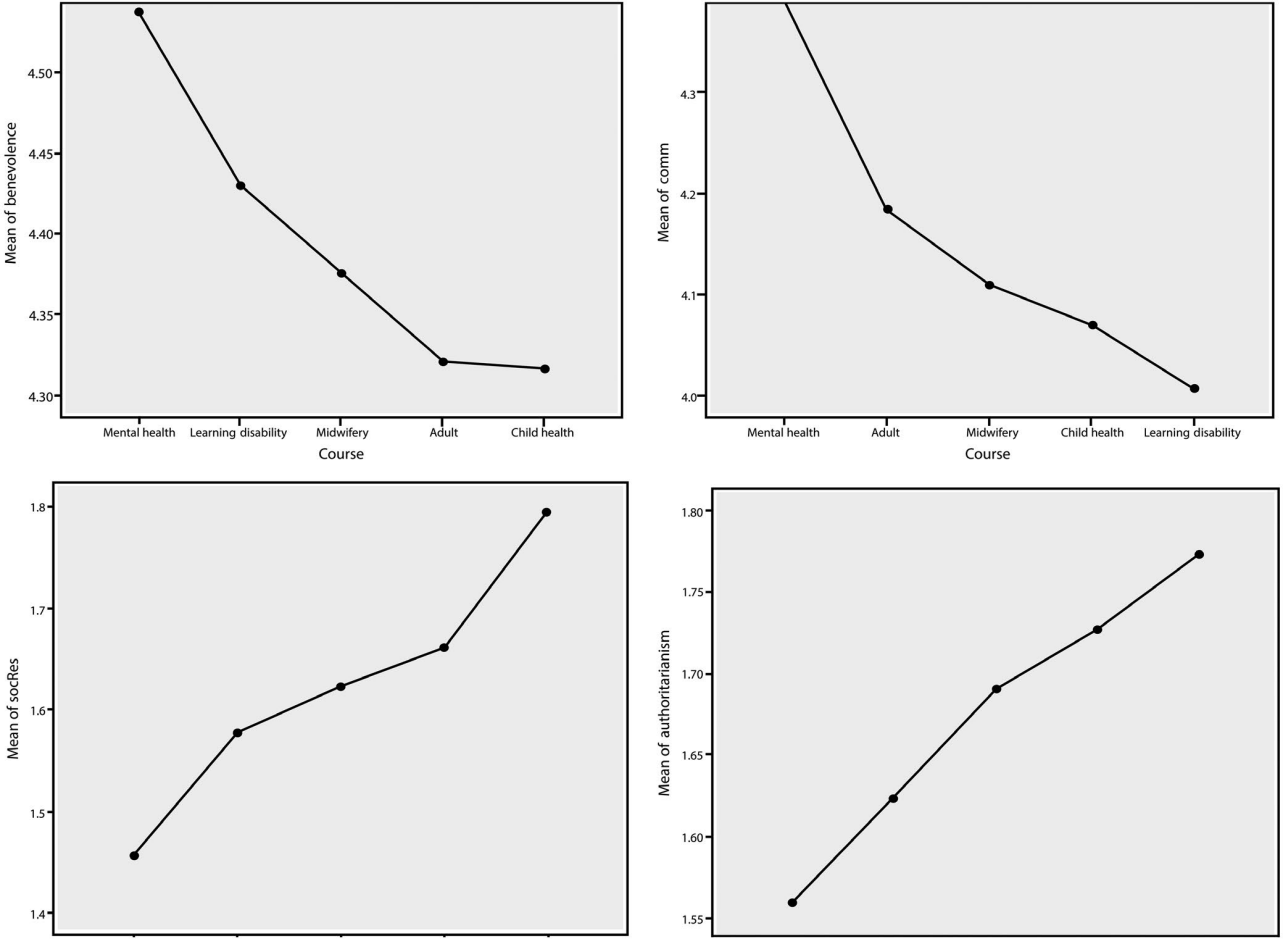
Means plots for Community Attitudes towards Mental Illness subscales by branch of nursing

From a potential population of 1,800 students, completed surveys were analysed from 437 (24%) participants, a reasonable return for a paper survey. Shih & Fan (2009) found that the average unweighted response rate of 39 comparative mail and electronic survey studies was 45%. Of the returns, 86 were adult nurses, 104 child health nursing, 70 learning disability nursing, 83 mental health nursing and 94 studying midwifery. Only fully completed questionnaires were included in the analysis. Participants were predominantly female (92%), consistent with the wider population, with ages ranging from 17–51 years old. Mean (*SD*) age was 25.6 years old, also broadly consistent with the whole population. CAMI subscale scores for each branch of nursing are illustrated in the means plot in Figure 1.

### 3.2 Hypothesis 1: Different branches of nursing and midwifery students will have different attitudes towards mental illness

One‐way Welch's ANOVAs were conducted for each subscale of the CAMI to explore whether there were statistically significant differences in the attitudes held by the different branches of nursing and midwifery. There were statistically significant differences at *p* < .05 level, across branches and between branches for each of the four CAMI subscales: authoritarianism, *F*(4, 432) = 4.55, *p* = .001, benevolence, *F*(4,432) = 5.39, *p* < .001, social restrictiveness, *F*(4,432) = 2.92, *p* = .021, and community mental health ideology, *F*(4,432) = 5.787, *p* < .001.

Post hoc comparisons using the Scheffe test indicated where the differences lay on each subscale. On the authoritarianism subscale, the mean score for child health nursing students (mean = 1.77, *SD* 0.38) was significantly higher than the mean score for mental health nursing students (mean = 1.56, *SD* 0.31). On the benevolence subscale, the mean score for mental health nursing students (mean = 4.54, *SD* 0.31) was significantly higher than the mean score for child health nursing (mean = 4.32, *SD* 0.35) and adult nursing students (mean = 4.32, *SD* 0.43). On the social restrictiveness subscale, the mean score for adult nursing students (mean = 1.66, *SD* 0.50) was significantly higher than the mean score for mental health nursing students (mean = 1.46, *SD* 0.34). Finally, on the community mental health ideology subscale the mean score for mental health nursing students (mean = 4.39, *SD* 0.49) was significantly higher than the mean score for child health nursing (mean = 4.07, *SD* 0.49), learning disability (mean = 4.01, *SD* 0.65) and midwifery students (mean = 4.11, *SD* 0.58).

### 3.3 Hypothesis 2: Previous exposure to people with mental illness will influence attitudes

One‐way between‐subject ANOVAs were conducted to compare the effects of personal experience rating on ratings on all four of the CAMI subscales.

Personal experience rating had a statistically significant effect, at the *p* < .05 level, on attitudes on all of the four CAMI subscales: authoritarianism, *F*(5, 431) = 4.29, *p* = .001, benevolence, *F*(5, 431) = 7.13, *p* < .001, social restrictiveness, *F*(5, 431) = 4.48, *p* = .001, and community mental health ideology, *F*(5, 431) = 7.10, *p* < .001.

Post hoc comparisons using the Scheffe test indicated that on the authoritarianism subscale, the mean score for students who rated themselves as 0, so having had no previous personal experience of mental illness (mean = 1.79, *SD* 0.35), was significantly higher than the mean score for those rated themselves as having had 4 (mean = 1.58, *SD* 0.33) or 5 (mean = 1.58, *SD* 0.37).

Post hoc comparisons also indicated that on the benevolence subscale, the mean score for students who rated themselves as having had 0 previous personal experience of mental illness (mean = 4.23, *SD* 0.41) was significantly lower than the mean score for those rated themselves as having had 4 (mean = 4.46, *SD* 0.34) or 5 (mean = 4.54, *SD* 0.33). The scores of those who rated themselves as a 3 (mean = 4.34, *SD* 0.37) were also significantly lower than those who rated themselves as 5 (mean = 4.54, *SD* 0.33).

Post hoc comparisons using the Scheffe test indicated that on the social restrictiveness subscale, the mean score for students who rated themselves as having had 0 previous personal experience of mental illness (mean = 1.74, *SD* 0.51) was significantly higher than the mean score for those rated themselves as having had 4 (mean = 1.51, *SD* 0.37) or 5 (mean = 1.48, *SD* 0.44).

Post hoc comparisons using the Scheffe test indicated that on the community mental health ideology subscale, the mean score for students who rated themselves as having had no previous personal experience of mental illness (mean = 3.91, *SD* 0.55) was significantly lower than the mean score for those rated themselves as having had 5 (mean = 4.41, *SD* 0.50). Those who rated themselves as having had 5 (mean = 4.41, *SD* 0.50) were also significantly higher than those who rated themselves as having had 3 (mean = 4.14, *SD* 0.58).

Professional experience rating also had a statistically significant effect, at the *p* < .05 level, on attitudes on all of the four CAMI subscales: authoritarianism, *F*(5, 431) = 2.93, *p* = .013, benevolence, *F*(5, 431) = 2.811, *p* = .016, social restrictiveness, *F*(5, 431) = 2.401, *p* = .036, and community mental health ideology, *F*(5, 431) = 5.47, *p* < .001.

Post hoc comparisons using the Scheffe test indicated that on the community mental health ideology subscale, the mean score for students who rated themselves as having had the maximum previous professional experience of mental illness, a score of 5 (mean = 4.40, *SD* 0.55), was significantly higher than the mean score for those rated themselves as having had 0 (mean = 4.06, *SD* 0.52) or 1 (mean = 3.58, *SD* 0.55).

### 3.4 Hypothesis 3: Each branch of nursing and midwifery students will have different attitudes towards mental illness throughout first year, second year and third year

No significant differences in any of the four subscales were found between first, second and third years studying mental health, child health, learning disability, adult nursing or midwifery. At the time that this study was conducted, some adults nursing students may have had professional experience of dementia during care of older people placements. Any branch or nursing or midwifery students may have also come across people with comorbid mental health issues during their placements. Otherwise experience of mental health would only be gained through “hub and spoke” experiences, for example by visiting a specialist service or ward for a few hours or a day.

## 4 DISCUSSION

Mental health student nurses in this study showed more positive attitudes to people with mental illness than any other branch of student nursing or midwifery. They showed significantly more positive scores across all four CAMI subscales (Figure 1). Results also showed that levels of stigma are a function of exposure. Those participants describing themselves as having had more personal or professional exposure to people with mental illness showed more positive attitudes than those without this exposure. This relationship held regardless of chosen field of study. There was no statistically significant change in attitudes from year to year in any branch of nursing or midwifery.

This suggests several related conclusions. First, students appear to be attracted to courses consistent with their existing values, attitudes and beliefs (Festinger, 1957). It would be expected and hoped that student mental health nurses have the most advanced attitudes in relation to mental illness and that is consistent with the findings here. Edward et al. (2015) noted that students entering the mental health nursing profession in both the United Kingdom and Australia were more likely to have worked in the specialty in another area. They also noted that previous exposure to mental health or psychiatry in a professional or personal context was more likely to make nursing students choose to work in the specialty. Thus, it is possible that nursing students who attend courses specifically dealing with “mental health” or “psychiatric” nursing are more likely to have less stigmatized views of these with a diagnosis of a mental illness. Secondly, regardless of beliefs on entry to nursing, stigma can be addressed by exposure to people with mental illness (Fokuo et al., 2017). Thirdly, the university studied here does not appear to provide this exposure, or if it did it was ineffective, because otherwise stigma scores would have shown a positive change year on year and they did not. These conclusions will be examined in more depth, but the most important caveat is that this was a cross‐sectional study and so any change over time cannot be inferred because the make‐up of years 1, 2 and 3 is different. To explore the impact of exposure over time, a longitudinal study would be needed (Parahoo, 2014).

Nevertheless, this snapshot revealed some important results. For example, both child and adult branches showed statistically significant differences in the benevolence subscale, in comparison with the mental health branch. This subscale represents the desire to “do good” towards people with mental illness according to Taylor and Dear (1981). Child health nursing students also showed a statistically significant difference in the authoritarianism subscale. Good practice in mental health values patient involvement and empowerment, encouraging people towards recovery (Grundy et al., 2016). Authoritarianism by contrast represents a more paternalistic view of mental health treatment and care (Tambuyzer, Pieters, and Van Audenhove, 2014).

However, although this contrast is supported by statistically significant differences, it is important to state that all sub‐groups’ average answers fell within the “positive” side of each subscale. This would suggest that child health nursing students do not generally hold views which could be described as “authoritarian.” They just held views which are more authoritarian than those of mental health nursing students.

Similar results are noted in the “community mental health ideology” sub‐group. This was shown to be significantly lower in child health nursing, learning disability nursing and midwifery than in mental health nursing. It is worth noting that none of the different population groups displayed views that could be characterized as “negative.” However, the results from the CAMI do indicate that the mental health nursing student population had views which could be categorized as “more positive” in comparison with child, learning disability and adult nursing and midwifery. The result may still be clinically relevant. Having some groups of nurses less ideologically committed to community care for people with mental illness may have clinical implications. International policy has seen a drive towards an increase in resources allocated to community teams in the past few decades (Rogers & Pilgrim, 2014), particularly in mental health (Thornicroft and Tansella, 2014). If this venture is to be successful in the long term, then it will be important not just for mental health nurses to feel positive about community care for people with mental illness. All nurses should support this, and university offers a perfect opportunity to challenge any stigma. The next section discusses the practicalities of this.

In terms of limitations, it is important to note that this study was completed at a United Kingdom University. The United Kingdom has a different style of nursing education as compared with most other countries (Stuhlmiller, 2005). Students in the United Kingdom choose a specialism before beginning their degree, whereas other countries generally provide a “general” nursing degree, with students choosing to complete further education in a speciality, postqualification (Lahtinen, Leino‐Kilpi, and Salminen, 2014). In this sense, the attitudes of prospective student nurses in non‐specialist education countries are less relevant than in the United Kingdom, as they do not choose what type of nursing they wish to pursue employment in until their formative education is complete. Thus, the findings from this study may be less relevant to student nurses out with the United Kingdom.

### 4.1 The importance of exposure

One of the other purposes of this study was to examine whether there would be a difference in attitudes according to level of both personal exposure and professional exposure to mental illness. Results support this hypothesis, consistent with a body of research that also show an increase in exposure to people with mental illness, is associated with an increase in positive attitudes (Happell, 2008; Henderson, Happell, & Martin, 2007; Schafer et al., 2011; Thongpriwan et al., 2015). More exposure to people with mental illness results in subjectively more positive attitudes.

On deeper analysis, it was found that although personal exposure and professional exposure to people with mental illness were both associated with positive attitudes, personal exposure was a stronger factor on all four subscales of the CAMI (Taylor & Dear, 1981). These findings support and encourage the recruitment of people with lived experience in the UK (Repper, 2014). Lived experience brings not only skills and knowledge that can be built on (Gilbert & Stickley, 2012), but as this research suggests more positive attitudes. The importance of this is highlighted in a review by Delaney (2012) who found that attitudes have the potential to affect the quality of nursing care provided.

Specifically, nurses with negative attitudes towards mental illness tend to feel that they cannot provide a sufficient quality of care (Reed & Fitzgerald, 2005), subsequently potentially affecting the therapeutic relationship between patient and nurse (Linden & Kavanagh, 2011). Although not as strong as personal exposure, professional exposure also resulted in statistically significant positive attitude scores on the CAMI. This suggests that undergraduate nursing and midwifery students would benefit from more exposure during their training than is currently the case.

Nevertheless, results from this study highlight the importance of mental health clinical placements as an essential part of nurse undergraduate training, not only for the development and honing of necessary nursing skills (Happell, 2008) but also as a means of fostering positive attitudes. This, in turn, has the potential to work towards decreasing inequality and discrimination in mental health care (Delaney, 2012; Hoekstra, Meijel, & Hooft‐Leemans, 2010).

As the regulatory body of nurses and midwives and those responsible for setting out preregistration standards, the Nursing and Midwifery Council (NMC, 2010) stipulates that all branches of student nurses and midwives should attempt to gain experience in multiple fields of nursing throughout their undergraduate training. However, there is currently no guidance on the amount of time required to be spent during cross‐field experiences. Currently, they have the potential to be completed within a one day's visit to a selected area, or even through “virtual placements” (Barrett & Jackson, 2013). These may not be enough to foster positive attitudes towards mental illness and may possibly be detrimental.

### 4.2 The J‐curve

On plotting the raw data, it was noted that there was an initial *drop* in CAMI scores from those who rated themselves as having no professional exposure to those who reported having a little. Positive attitudes were then observed to increase as the amount of exposure increased. This trend, although not statistically significant, could be clinically relevant. It would suggest that brief, one‐off cross‐field experiences have the potential to leave student nurses and midwives with more negative attitudes than they began with.

This would be expected in the literature on organizational change management. In particular, the J‐curve would predict exactly this: a small dip in performance until the participant felt increasing comfort with the new and improved venture or process (Snowden & Kolb, 2016). Further research should explore the generalizability of this trend and if there is an optimal level of exposure that would see an increase in positive attitudes. This research would start from the perspective that very short amounts of exposure are likely to have the opposite effect to the one intended.

As well as the time spent having the exposure, it is intuitive to conclude that the *type* of exposure would be very important; it is unlikely to simply be a case of “more is better.” The quality and purpose of the exposure is essential as Linden and Kavanagh (2011) found when mental health nurses who had prolonged exposure to acutely unwell inpatients were more likely to have more negative attitudes than to those working in other areas of mental health, such as in the community. Prolonged exposure to acute mental illness and crisis, along with the associated violence and aggression, has been shown to be detrimental to student nurse development (Martensson, Jacobsson, & Engstrom, 2014).

The current research also did not find a statistically significant effect of participants’ overall experience of their exposure to mental illness. In other words, it did not seem to matter whether they perceived their experiences of mental illness as either positive or negative (measured via a self‐reported Likert scale). This is in contrast to previous research which has found that positively perceiving experiences of mental health placements aids in fostering positive attitudes (Happell, 2008).

### 4.3 Limitations

The CAMI (Taylor & Dear, 1981) consists of direct statements that can be construed as divisive and outdated, consistent with its development in 1981. The concept of “mental illness” is now considered paternalistic and reminiscent of a darker era (Snowden, 2009). However, a review by Ross and Goldner (2009) found that similar statements to those in the CAMI were mirrored by nurses expressing stigmatized attitudes towards mental illness. Research by Hoekstra et al. (2010) also mirrored similar comments among their participants. Therefore, this measurement tool would unfortunately still seem fit for purpose in this study. The divisive nature of the statements may also have helped to prevent ambiguity around the meaning of the statement and thus providing more accurate responses. The four‐factor structure claimed by Taylor and Dear (1981) was not tested in this study. There is therefore the risk that the scale may not be measuring what it claims to.

Another small but noteworthy limitation could be that the four principle researchers of this study were also student mental health nurses in their second year on the nursing course at the same HEI at time of data collection. However, the risks of biases, particularly in the second‐year branch of mental health nurse students, were eliminated in the same way that other biases were; participants were fully informed of the study before participation, questionnaires were anonymized, and participants could opt out at any time.

Perhaps one of the biggest limitations of this study is that, despite providing a useful and timely exploration of student nurse and midwife attitudes towards mental illness, it is only a single, brief snapshot, and therefore, its generalizability should be considered cautiously. A longitudinal study would undoubtedly offer a more accurate investigation into the way attitudes change throughout undergraduate training and clinical placements.

### 4.4 What this study adds and implications

The results and implications from this study should be considered tentatively given its limitations. However, this research does provide an insight into the attitudes that may be prevalent throughout nurse training and this, in itself, presents a foundation on which to work towards further research on fostering positive attitudes.

This study provides the only research on attitudes of nursing students across *all* branches of nurse training in the UK, including child, adult, learning disability, midwifery and mental health. Therefore, this study provides the first snapshot of the attitudes that undergraduate student nurses and midwives may hold and what may be shaping and forming these attitudes.

Undoubtedly, there is a complexity of other factors at play but what this study highlights is that exposure may be central in shaping attitudes. It would therefore seem pertinent that all branches of nursing and midwifery and not just mental health have clinical placements in mental health areas. What this study also adds is that this exposure needs to be meaningful in duration, or it can have the opposite effect to the one intended. A little exposure seems to reinforce negative views. This is particularly important to know at a time when nurse education in the UK is preparing to demand ever more of its students (Nursing and Midwifery Council, 2018). The temptation will be for curriculum planners to provide short or even “virtual” learning experiences for non‐mental health students so that adult, child, learning difficulties or midwifery students can sign off the requisite competence in mental health. This study has shown that this could be a mistake and curriculum planners may run the risk of reinforcing negative beliefs, exactly the opposite of the intended aim.

This study suggests that there may be a responsibility for curriculum planners to ensure, as far as possible, that placements are tailored to student nurse and midwifery courses to aid in the fostering of positive attitudes towards mental illness. That is, placements are optimal in terms of type and length. For example, non‐acute areas for non‐mental health student nurses unlikely to experience further mental health placements to combat any negative attitudes being created and longer duration of placements to reduce J‐curve effects. The study would also suggest extending the drive to recruit people with lived experience onto undergraduate mental health nursing courses to that of *all* branches of nursing.

## 5 CONCLUSION

In conclusion, students choosing to study mental health nursing hold more sophisticated views about stigma than their adult, child, learning difficulties and midwifery colleagues. This corroborated Schafer et al. (2011) earlier work studying student adult and mental health nurses but also extended it to include all branches of student nursing and midwifery. This suggests that the students choosing mental health nursing have chosen it because their values are already well developed in this area. This is important to know, but the more interesting question is why they came to hold those values in the first place.

The answer is likely to be exposure. That is, the study showed the positive relationship between personal and professional exposure to mental illness and reduction in stigma. So, while it appears less stigmatizing students are selecting mental health nursing as their degree, this study also showed that stigma can be mitigated in *any* branch of nursing through exposure. This study would suggest exposure in mental health areas that are optimal in both type and duration. The optimal duration of exposure in this area should be a focus of future research.

The future of mental health care concerns everyone. There is a need to challenge negative attitudes and foster positive attitudes towards mental health. If this can be achieved at the beginning of a nursing or midwifery career, it has the potential to destigmatize people with mental illness (Delaney, 2012; Martensson et al., 2014; Schafer et al., 2011). This, subsequently, can promote a better understanding and appreciation of mental health more generally and aid effective multidisciplinary team working and the provision of quality holistic care.

## CONFLICT OF INTEREST

The four principle researchers of this study were also student mental health nurses in their second year on the nursing course at the same HEI at time of data collection. The two other researchers were also faculty at the same HEI. However, the risks of biases, particularly in the second‐year branch of mental health nurse students were eliminated in the same way that other biases were; participants were fully informed of the study before participation, questionnaires were anonymized, and participants could opt out at any time.

Permission to carry out the study was given by the Edinburgh Napier University School of Health and Social Care Ethics Committee. All participants were provided with information about the study prior to taking part, were told that their data would be analysed anonymously, signed consent forms and had the option to opt out at any time.
